# A novel seven-gene signature as Prognostic Biomarker in Hepatocellular Carcinoma

**DOI:** 10.7150/jca.44573

**Published:** 2020-07-31

**Authors:** Hui Xie, Shouping Liu, Ziying Zhang, Peng Chen, Yongguang Tao

**Affiliations:** 1 Hunan Cancer Hospital and The Affiliated Cancer Hospital of Xiangya School of Medicine, Central South University.; 2 Key Laboratory of Carcinogenesis and Cancer Invasion, Ministry of Education, Department of Pathology, Xiangya Hospital, Central South University, Hunan, 410078 China.; 3 NHC Key Laboratory of Carcinogenesis (Central South University), Cancer Research Institute and School of Basic Medicine, Central South University, Changsha, Hunan, 410078 China.; 4 Department of Thoracic Surgery, Hunan Key Laboratory of Early Diagnosis and Precision Therapy in Lung Cancer, Second Xiangya Hospital, Central South University, Changsha, 410011 China.; 5 Department of Oncology, Third Xiangya Hospital, Central South University, Changsha, Hunan, 410013, China.; 6 Department of Urology, Xiangya Hospital, Central South University, Changsha, Hunan, 410008, China.

**Keywords:** hepatocellular carcinoma, prognosis, risk score, prognostic signature

## Abstract

Purpose: Our study is designed to develop and certify a promising prognostic signature for hepatocellular carcinoma (HCC).

**Materials and methods:** We retrospectively analyzed mRNA expression profiles and clinicopathological data fetched from The Cancer Genome Atlas (TCGA) and Gene Expression Omnibus (GEO) datasets. We formulated a prognostic seven-gene signature composed of differentially expressed mRNAs (DEmRNAs) between HCC and nonneoplastic tissues through univariate Cox regression analysis. The receiver operating characteristic (ROC) curve, survival analysis and multivariate Cox regression analysis as well as nomograms were utilized to assess the prognostic performance of the seven-gene signature.

**Results:** The risk score based on a seven-gene signature categorized HCC subjects into a high- and low-risk group. There was significantly discrepant overall survival (OS) between patients in both groups and the corresponding ROC curve revealed a satisfactory predictive performance in HCC survival in both TCGA and GSE76427 cohort. Multivariate Cox regression analysis demonstrated that a seven-gene signature was an independently prognostic factor for HCC. Nomograms combining this prognostic signature with significant clinical characteristics conferred a crucial reference to predict the 1-,3- and 5 years OS.

**Conclusions:** Our study defined a promising seven-gene signature and nomogram model to forecast the OS of HCC patients, which is instrumental in clinical decision and personalized therapy.

## Introduction

Liver cancer is the second most frequent reason for tumor-associated deaths, with an approximated 841, 080 new cases and 780 thousand deaths occurring globally in 2018. In particular, almost one-half of new diagnoses and deaths are Chinese 1-3. HCC, accounting for almost 90% of all primary liver cancers, can be induced by multifarious risk factors, including hepatitis virus infection, metabolic disorders, aflatoxin and autoimmune hepatitis 1, 4. Despite recent encouraging progress in therapeutic interventions (such as surgery, radiofrequency ablation, targeted therapy, and radiotherapy), HCC patients display an unsatisfactory prognosis with a lower than 40% 5-year survival rate because of frequent recurrence or distant metastasis 5-7. Several clinicopathological parameters, including pathologic differentiation, tumor-node-metastasis (TNM) staging and vascular invasion, constitute conventional prognostic models to predict the OS of HCC patients 8. Nevertheless, their predictive performance is not very encouraging owing to the great heterogeneity of HCC. Additionally, regarding several valuable biomarkers, such as des-γ-carboxyprothrombin 9 and alpha-fetoprotein (AFP) 10, their prognostic efficiency is variable among studies, which is partly ascribed to difference in sample size, assay methods or statistical methods 6.

Recent advance in genome-sequencing technologies has investigated the prognostic prediction of gene signatures in HCC. A majority of studies have concentrated on single gene molecule at mRNA level 11-17. However, multiple-gene-based signatures are more robust to evaluate HCC prognosis compared with the predictive power of single-biomarker. For example, the prognostic predictive value of apolipoprotein A1 (*APOA1*) combined with C-reactive protein (CRP) is more favorable than that of serum AFP alone 12. Sulfite oxidase (*SUOX*) integrated with AFP are sufficient to predict the performance and recurrence risk of HCC 18. Similarly, Dong et al demonstrated that synergic analysis of *STAT* genes (*STAT5A*, *STAT5B* and *STAT6*) exhibited a more desirable predictive efficiency for HCC prognosis than did single gene 19. The accumulating accessibility of genome-wide gene expression information in HCC potentially permits the development of a credible gene signature 20, 21. Thus, deep excavation of publicly accessible genomic data is a considerable strategy to appraise novel multi-gene signatures with reliable predictive power in the OS of HCC patients, thus conferring promise to improve patients' risk stratification and individual therapeutic interventions. In our study, we acquired the HCC mRNA expression profile from the TCGA and GEO to formulate a prognostic seven-gene signature and a nomogram with satisfactory credibility for HCC patients, which is conducive to covering the imperfection of the current staging system.

## Materials and Methods

### Data extraction and manipulation

The original mRNA expression information was acquired from TCGA and GEO database, respectively. HCC patients without crucial clinical information (including follow-up or survival status) or mRNA expression data were excluded. The DEmRNAs were identified between tumor tissues and normal samples. We normalized the RNA expression data through multi-array average (RMA) expression measure method. We further investigated DEmRNAs through DESeq vesion 1.38.0 R package in TCGA dataset and by the Limma version 3.36.2 R package in GEO dataset 22. We further utilized univariate Cox regression analysis to extract DEmRNAs that were significantly related to the prognosis of HCC patients. The hazard ratio (HR)-cutoff value is conventionally set at 1 to define protective genes (HR < 1) and risky genes (HR > 1). We conducted a crosstalk between above two datasets and eventually selected seven reliable DEmRNAs associated with the OS of HCC.

### Construction of prognostic signature

A prognostic risk score model was further formulated in accordance with the mRNA expression levels and its corresponding regression coefficient (β). The calculative method was as follows: risk score = the expression level of transketolase (*TKT*) * β*_TKT_*+ the expression of *TTC39B* * β*_TTC39B_* + the expression level of poly-N-acetyllactosamine (*PLN*) * β*_PLN_* + the expression level of *CBFA2T2* * β*_CBFA2T2_* + the expression level of heat shock protein beta 3 (*HSPB3*) * β*_HSPB3_* + the expression level of Progestin and adipoQ receptor 4 (*PAQR4*) * β*_PAQR4_* + the expression level of *C21orf58* * β*_C21orf58_*. All incorporated HCC patients were categorized as high- and low-risk groups on the basis of a median risk score.

### Establishment of nomogram

Nomogram has a robust capacity to predict tumor prognosis 23, 24. A nomogram was established through incorporating all significant prognostic clinicopathological parameters determined through multivariate Cox regression analysis, thus estimating the probability of 1 -, 3-, and 5 years-OS of HCC. We calculated the concordance index (C-index) to identify the discrimination of a nomogram. The calibration curve of a nomogram was utilized to vividly assess the consistency between its prediction probabilities and the actual observation.

### Statistical analysis

To evaluate the survival differences between low- and high-risk HCC patients, we performed survival analysis through Kaplan-Meier curve combined with the log-rank test. We also conducted univariate and multivariate Cox regression analyses to identify the association between OS and risk score as well as clinicopathological features. The ROC curve with the corresponding area under the curve (AUC) was rendered to estimate the predictive performance of the prognostic gene signature for HCC survival by the R package “survival ROC” 25. *P* < 0.05 was defined as statistically significant.

## Results

### Recognition of differentially expressed genes associated with prognosis

Based on TCGA dataset, 3256 DEmRNAs were identified in HCC samples (n = 374) when compared with noncancerous samples (n = 50). Similarly, a total of 12674 DEmRNAs were extracted from GSE47595 (both P < 0.05). The heatmap of the DEmRNAs was revealed in **Supplementary Figure 1A and 1B**. We further utilized univariate Cox regression model to identify 1569 DEmRNAs from TCGA and 174 DEmRNAs from GSE47595, which were all significantly related to OS of HCC patients (P < 0.05). To narrow down the range of genes, seven differentially expressed genes associated with HCC prognosis were confirmed by overlapping the above two datasets (**Supplementary Figure 1C**) and were further incorporated into a prognostic gene-signature. Collectively, the seven genes were as follows: *TKT*, *TTC39B*, *PLN*,* CBFA2T2*,* HSPB3*, *PAQR4*, *C21* or* f58.* The general characteristics of the seven genes were summarized in** Table 1**. Under the condition of the cutoff value of HR = 1, there were four common candidate risky genes (*TKT*, *CBFA2T2*, *PAQR4*,* C21orf58*) and three candidate protective genes (*TTC39B*, *PLN*,* HSPB3*) (**Table 2**).

### Constitution and validation of a seven-gene prognostic signature

**Figure 1:**
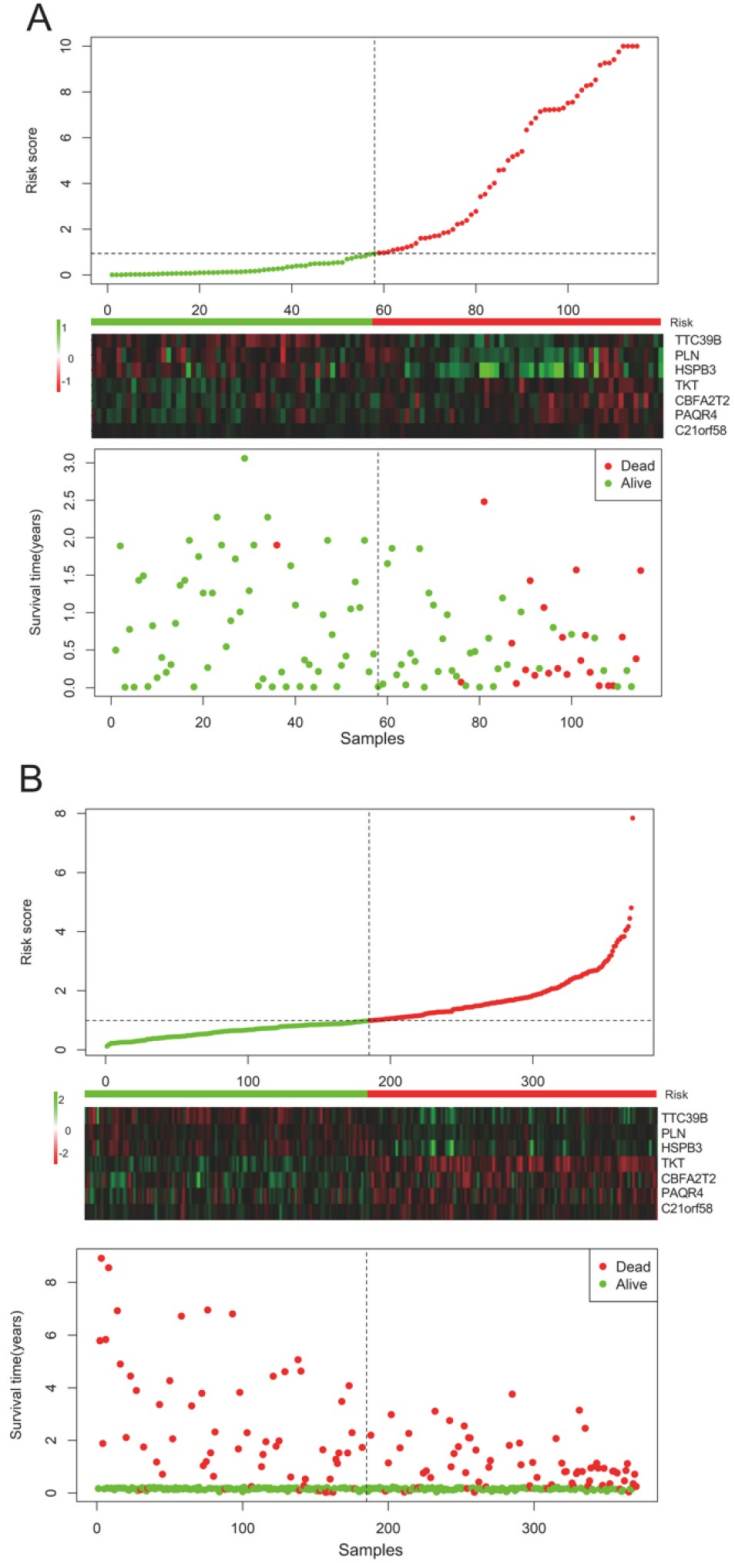
Risk-score analysis of HCC patients in the (**A**) TCGA and (**B**) GSE76427 datasets. There was a graphical representation concerning the risk score distribution, gene expression profiles, and survival condition of HCC patients.

**Figure 2:**
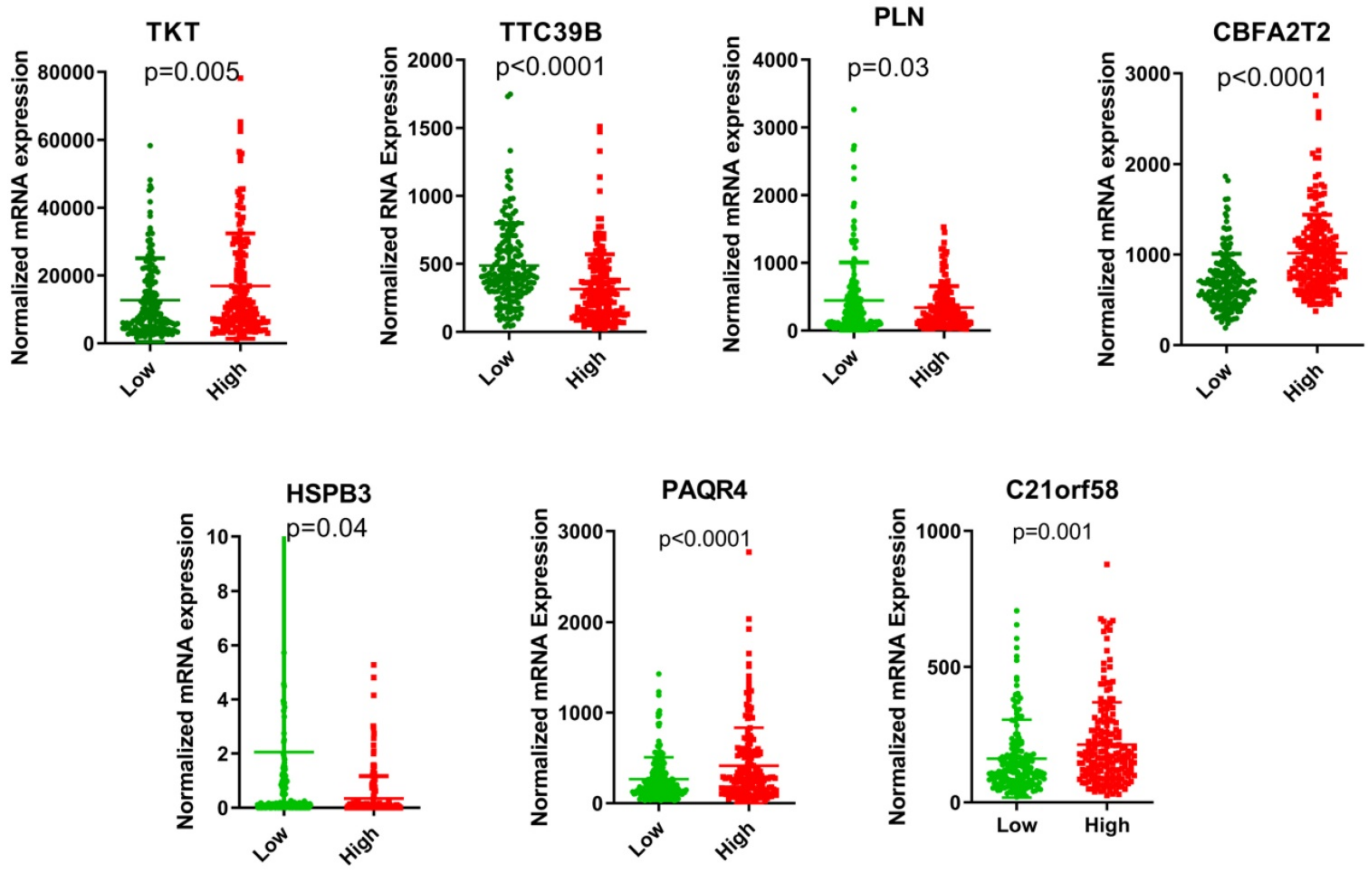
The expression levels of the seven genes based on TCGA cohort in the risk groups.

**Figure 3:**
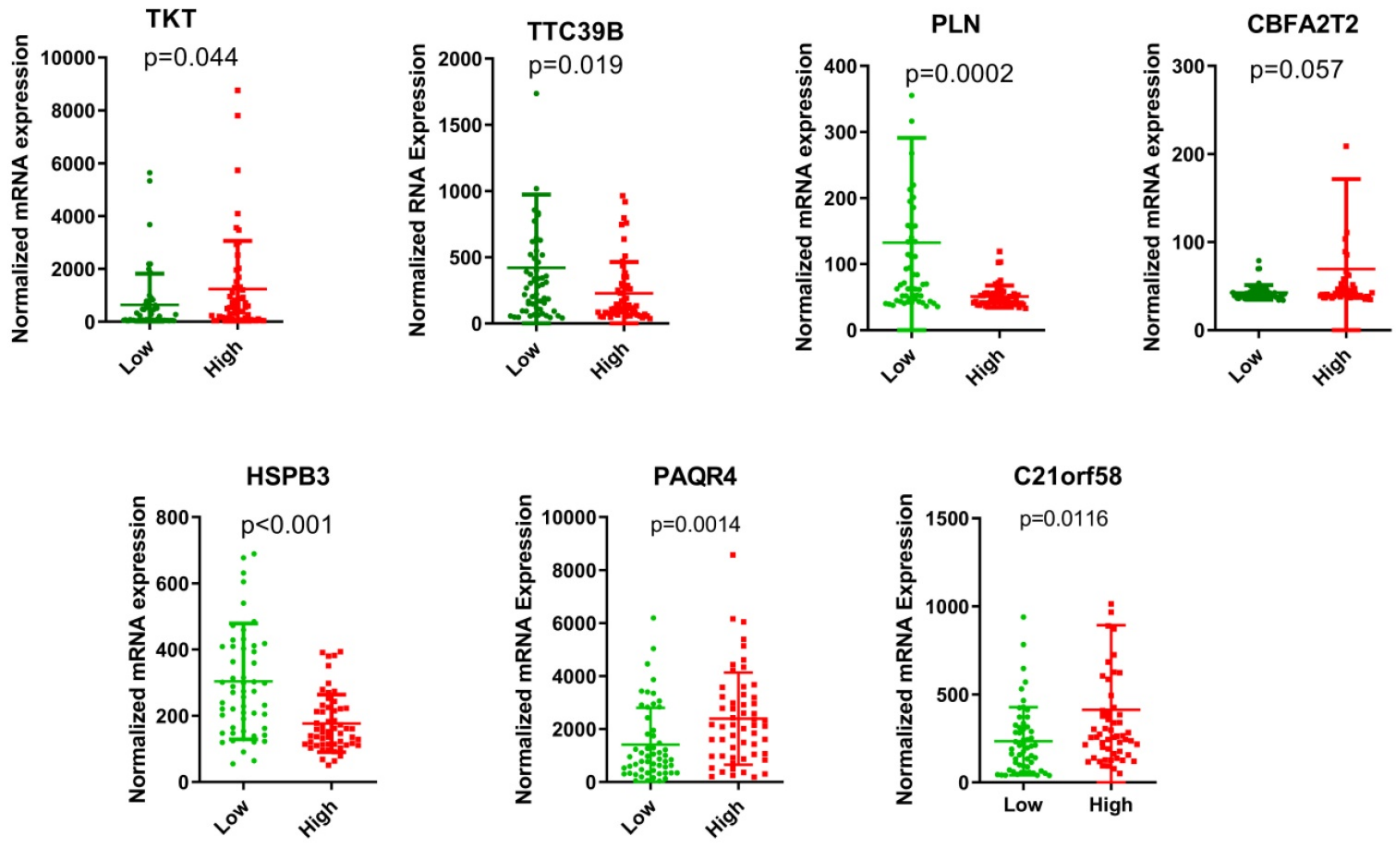
The expression levels of the seven genes based on GSE76427 cohort in the risk groups.

The risk score of each HCC patient was calculated based on the equation: risk score = (0.345) * *TKT* value + (0.213) * *CBFA2T2* value + (0.132) * *PAQR4* value + (0.115) * *C21orf58* value + (-0.015) * *TTC39B* value + (-0.043) * *PLN* value + (-0.077) * *HSPB3* value. All HCC subjects were stratified into high- and low-risk groups in accordance with risk score. The risk score distribution, gene expression and survival status of each HCC patient were revealed in **Figure 1**. For the seven genes, four genes corresponded to high risk (*TKT*, *CBFA2T2*, *PAQR4*, *C21orf58*; HR > 1) and three genes seemed to be protective (*TTC39B*,* PLN*,* HSPB3*; HR < 1). We made a comparison in the expression discrepancies of seven genes between high- and low-risk groups. Indeed, risky genes were prone to express in patients with high-risk scores, while patients in the low-risk group were characterized with protective genes expression (**Figure 2 and Figure 3**). To identify the relationship between risk score model and clinicopathological characteristics in HCC patients, we further analyzed the risk score level in HCC patients at different clinical stages. As revealed in **Table 3**, risk score based on this seven-gene prognostic model was significantly associated with N classification, M classification, histologic grade, AJCC staging, fibrosis score (both *P* < 0.05). Thus, these findings highlight that the level of risk score is related to diverse crucial pathological characteristics of HCC patients.

### Correlation between a seven-gene prognostic signature and HCC survival

**Figure 4:**
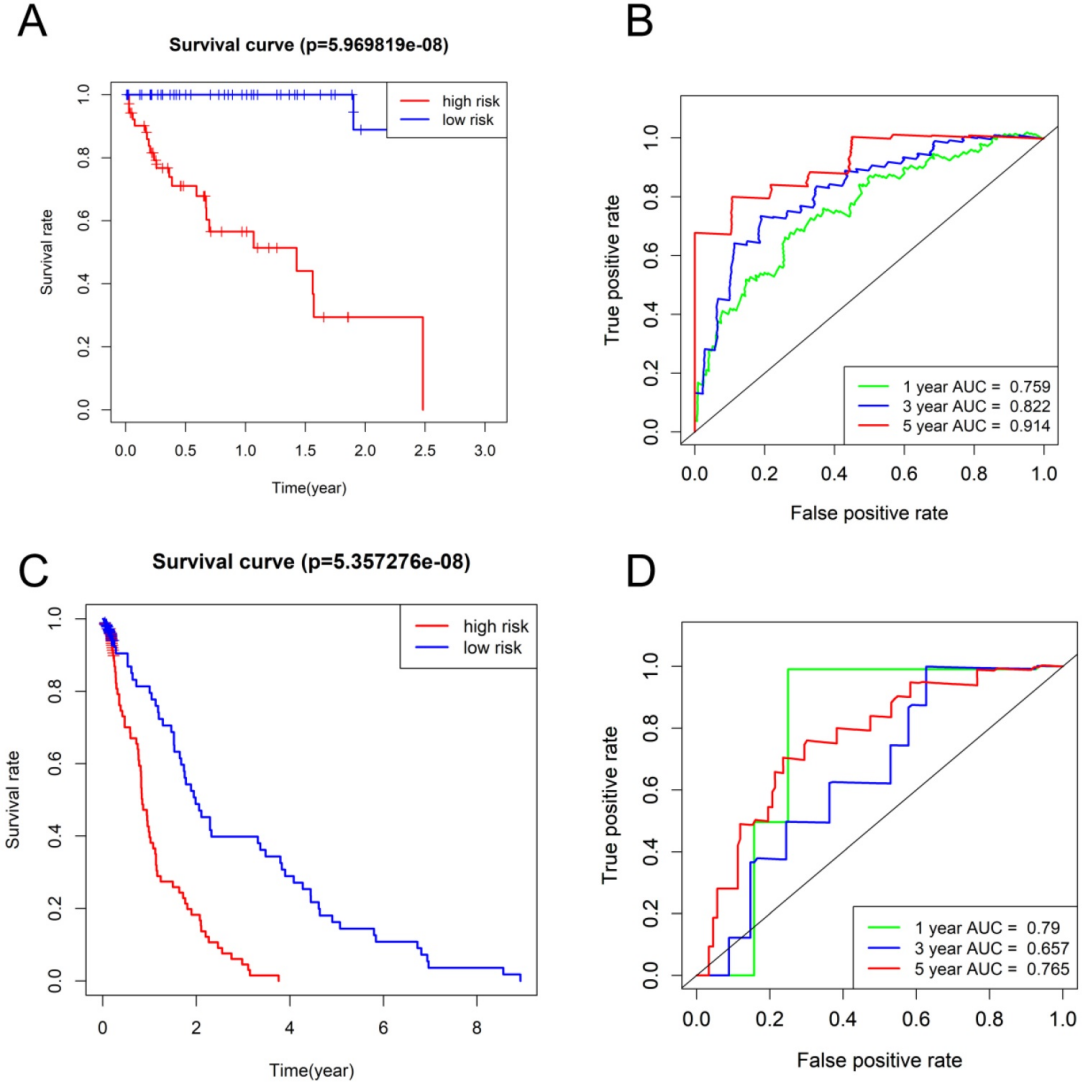
Survival analysis and time-dependent ROC analysis in (**A and B**) TCGA database and (**C and D**) GSE76427.

The HCC subjects in low-risk group were characterized with more satisfactory OS in comparison to those in high-risk group (*P* < 0.001) (**Figure 4A, C**). The time-dependent ROC curves based on TCGA dataset displayed that the AUCs for 1-year, 3-year, and 5-year OS were 0.759, 0.822 and 0.914, respectively and the corresponding values based on GSE76427 dataset were 0.79, 0.657, and 0.765, respectively (*P* < 0.05) (**Figure 4B, D**), highlighting a substantially effective predictive performance of the seven-gene signature for HCC prognosis.

**Figure 5:**
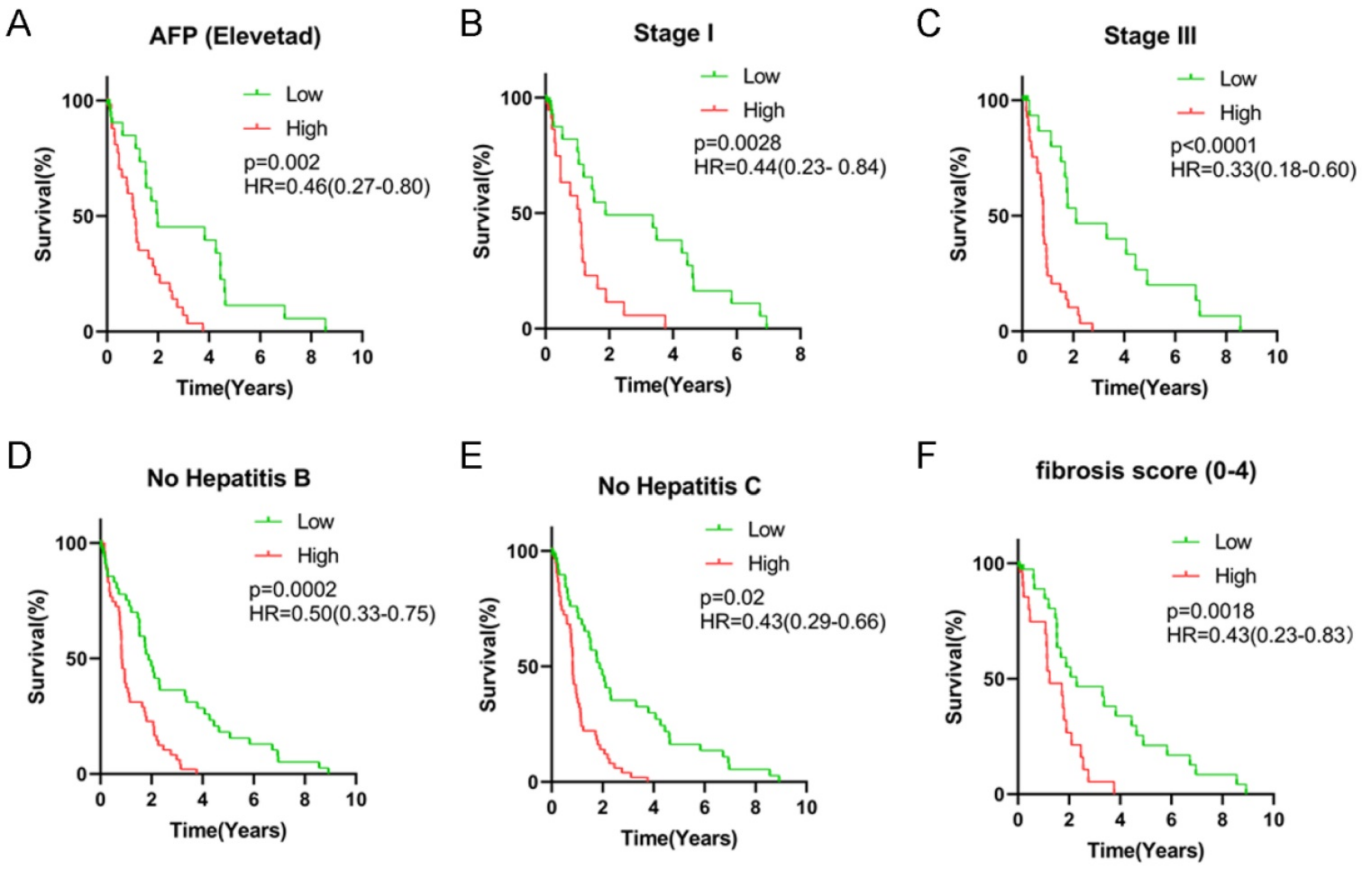
Survival analysis of risk score levels in different subgroups of HCC patients. OS analysis of (**A**) elevated AFP level, (**B**) TNM stage I, (**C**) TNM stage III, (**D**) no HBV infection, (**E**) no HCV infection and (**F**) fibrosis score.

**Figure 6:**
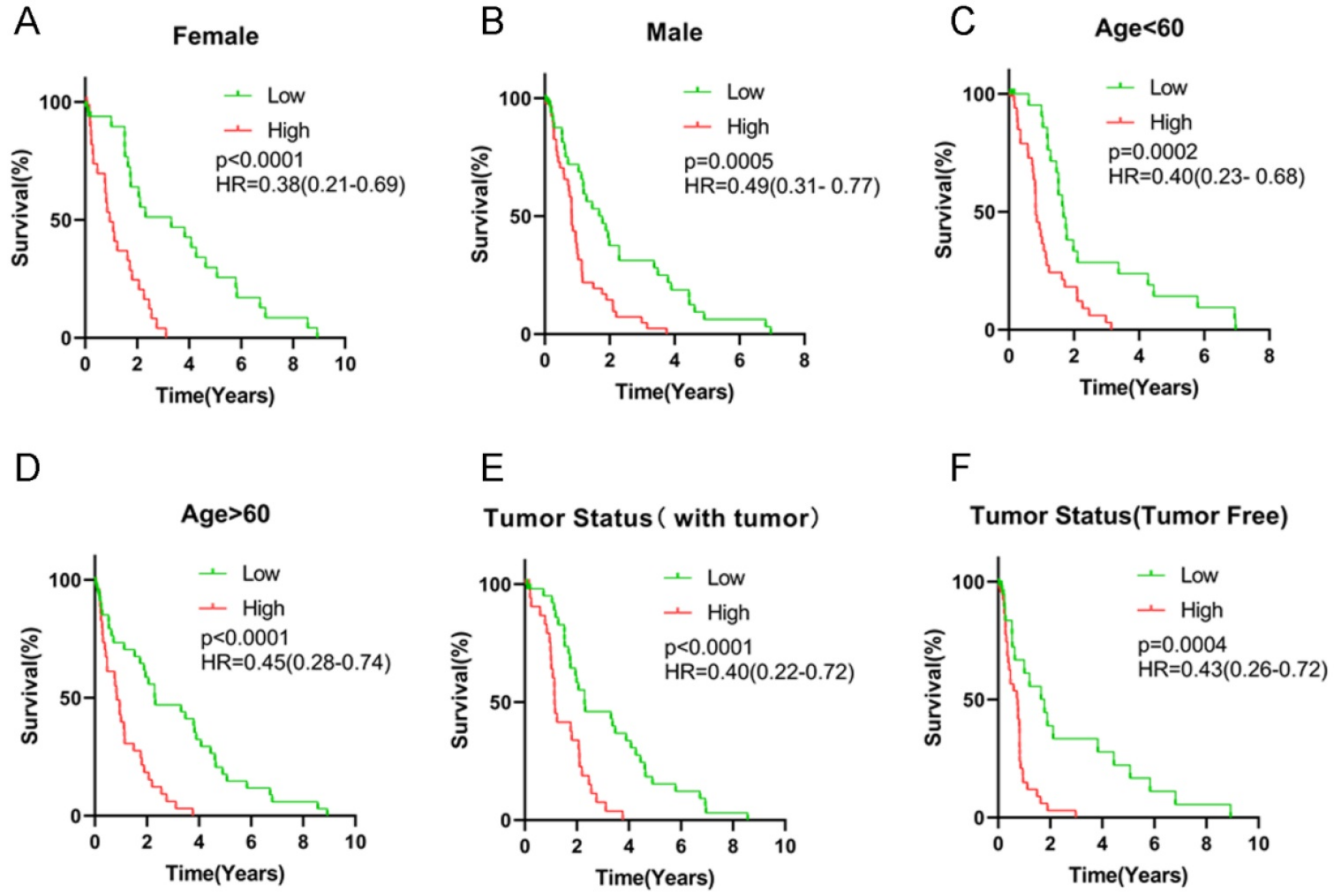
Survival analysis of risk score levels in different subgroups of HCC patients. OS analysis of (**A-B**) gender, (**C-D**) age, (**E-F**) tumor status.

All HCC subjects were further stratified into different subgroups based on clinicopathologic characteristics to confirm the association between signature risk score and HCC prognosis. Survival analysis revealed that regardless of age, gender and tumor status, there was statistically significant discrepancy in HCC prognosis between the high- and low-risk groups (HR = 0.38, 95% CI = 0.21-0.69, *P* < 0.0001 for female patients; HR = 0.49, 95% CI = 0.31-0.77, *P* = 0.0005 for male patients; HR = 0.40, 95% CI = 0.23-0.68, *P* = 0.0002 for patients with age < 60 years old; HR = 0.45, 95% CI = 0.28-0.74, *P* < 0.0001 for patients with age ≥ 60 years old; HR = 0.43, 95% CI = 0.26-0.72, *P* = 0.0004 for patients with free tumor; **Figure 5**). Low signature risk scores were significantly related to more favorable OS in the subgroup of patients with elevated AFP levels (HR = 0.46, 95% CI = 0.27-0.80, *P* = 0.002), at TNM stage I (HR = 0.44, 95% CI = 0.23-0.84, *P* = 0.0028) or stage III (HR = 0.33, 95% CI = 0.18-0.60, *P* < 0.0001), with low level of fibrosis (HR = 0.43, 95% CI = 0.23-0.83, *P* = 0.0018), without HBV infection (0.50, 95% CI = 0.33-0.75, *P* = 0.0002) or HCV infection (HR = 0.43, 95% CI = 0.29-0.66, *P* = 0.02) (**Figure 6**). Nevertheless, for HCC patients with HBV or HCV infection, normal AFP levels and at TNM stage II and IV, the statistically significant prognostic difference was not revealed between the high- and low-risk groups. Above findings imply that the risk score signature potentially serves as a robust prognostic marker for HCC patients and fails to be affected by parameters that usually trigger variations in the performance of conventional biomarkers. Moreover, as shown in **Supplementary Figure 2**, the predictive accuracy of our model was relatively satisfactory than additional clinical indicators, such as TNM stage, BCLC stage and pathological differentiation.

### Cox proportional hazards regression analysis

We further evaluated the effect of the seven-gene prognostic signature on the OS of HCC patients through univariate and multivariate Cox regression. For the whole TCGA cohort, univariate Cox regression revealed that gender, histologic grade, AJCC stage and fibrosis score as well as risk score were significantly correlated with HCC survival (both *P* < 0.05). The corresponding multivariate Cox regression analysis demonstrated that HCC patients with poor histologic grade, advanced AJCC stage as well as high risk scores potentially exhibited more unsatisfactory prognosis (both *P* < 0.05) (**Table 4**). Based on data extracted from GSE76427 database, BCLC stage, TNM stage and risk score were significantly associated with HCC prognosis through univariate and multivariate Cox regression analysis (both *P* < 0.05) (**Table 5**). Therefore, low risk score was indeed an independent protective indicator for HCC prognosis.

### Establishment and validation of a predictive nomogram

**Figure 7:**
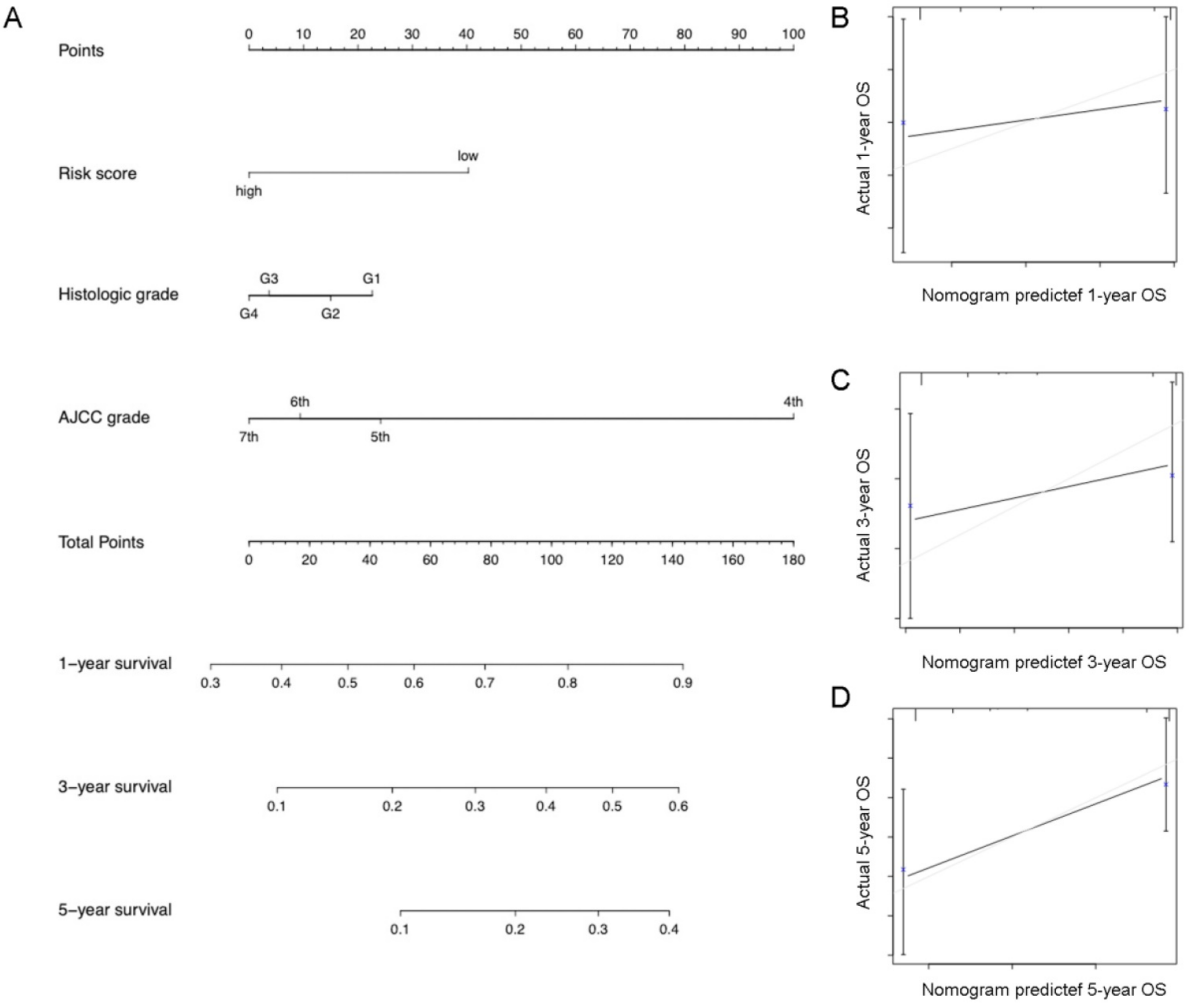
Nomogram combining risk score with significant clinicopathologic features to predict the OS of HCC patients in the TCGA dataset.

**Figure 8:**
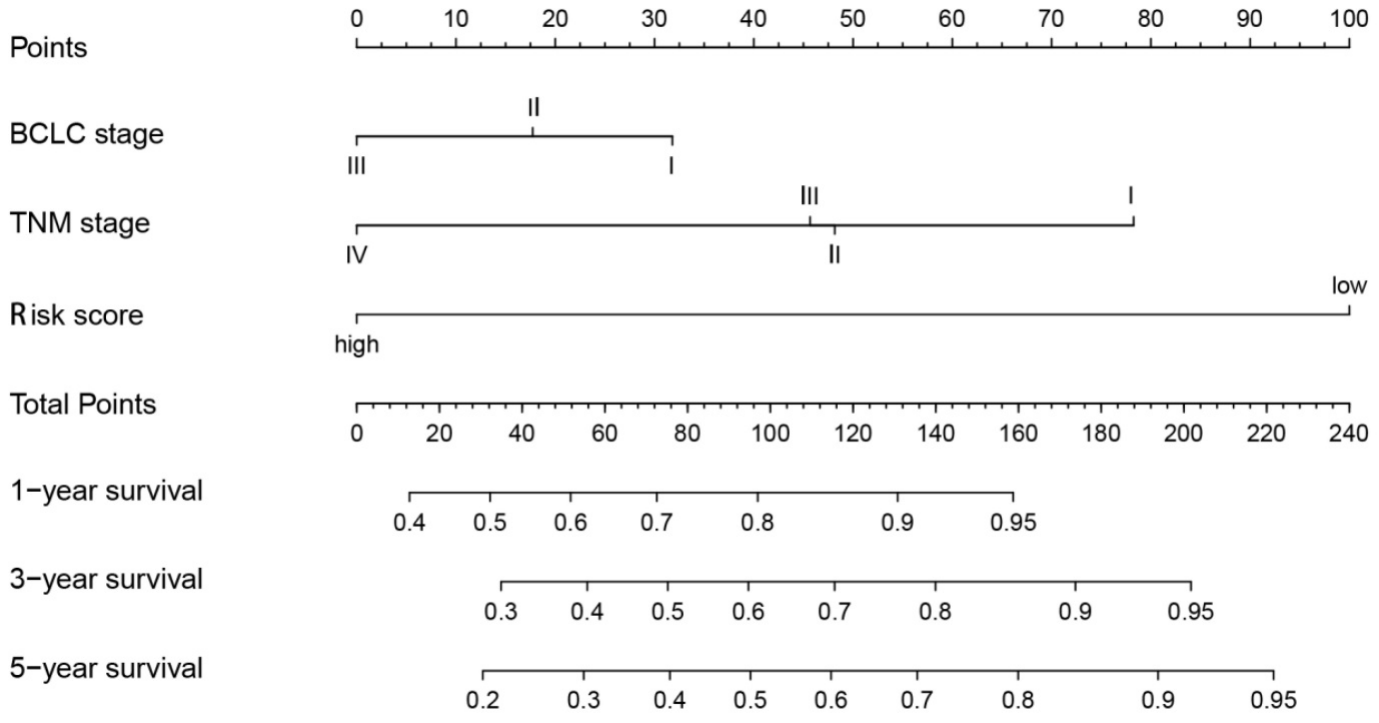
Nomogram combining risk score with significant clinicopathologic features to predict the OS of HCC patients in the GSE76427 dataset.

Eventually, we incorporated all significantly prognostic factors based on multivariate Cox regression analysis to formulate a nomogram, thus predicting OS of 342 HCC patients from TGGA. Specifically, AJCC stage and predictive risk score made the greatest contributions to HCC prognosis, followed by histologic grade in TCGA database (**Figure 7**). Risk score, TNM stage and BCLC stage exerted a crucial effect on the HCC prognosis in GSE76427 database (**Figure 8**). The C-index of nomogram associated with TCGA and GSE76427 database was 0.745 (95% CI: 0.676-0.816) and 0.7645 (95% CI: 0.699-0.835), highlighting a desirable predictive value of our nomogram models.

## Discussion

Accumulating studies have highlighted that genetic changes and defects in the signaling pathways exert a crucial effect on tumorigenesis and development of HCC, indicating the potential prediction value of molecular biomarkers in HCC prognosis 26. Furthermore, the prognostic gene signature combined with traditional clinical indicators exhibit a more satisfactory predictive performance than a single parameter 12, 27. Currently, multi-gene signatures based on abnormal mRNA levels have attracted much consideration and displayed a promising predictive potential in HCC prognosis 19, 28-30.

In our report, we selected the seven common genes (*TKT, TTC39B, PLN, CBFA2T2, HSPB3, PAQR4, C21* or* f58*) most significantly related to HCC prognosis through overlapping the TCGA and GEO databases. Each gene was defined as a risky gene (HR > 1) or protective gene (HR < 1) through univariate Cox regression analysis. A signature risk score based on the nine genes was developed and it conferred a standard to stratify HCC patients into high- and low-risk groups. We further conducted univariate and multivariate Cox regression analysis to validate the independent prognostic effect of this seven-gene signature on HCC. Kaplan-Meier curves showed that HCC patients in the high-risk group were characterized with unfavorable prognosis. Moreover, we exploited a nomogram with robust predictive performance to estimate survival through combining the signature risk score and additional clinicopathological characteristics with statistically significance. These findings highlight that the risk score is a stable, independent prognostic indicator with significant and effective predictive value for HCC patients based on our seven-gene model.

The mRNA *TKT* was one of the seven-gene prognostic signature in our study.* TKT*, a vital enzyme in the pentose phosphate pathway (PPP), is essential for tumor proliferation on account of its capability to influence NAPDH generation to counteract oxidative stress. Disturbing the redox homeostasis of cancer cells by genetic knockdown or pharmacologic inhibition of *TKT* sensitizes cancer cells to existing targeted therapy (Sorafenib) 31. Reduced expression of *TKT*, which regulate flux into pyrimidine biosynthesis, correlates with better prognosis in pancreatic cancer patients on fluoropyrimidine analogs 32. Specifically, *TKT* can promote the development of HCC in a non-metabolic manner via its nuclear localization and EGFR pathway 33. Loss of *TKT* in the liver increased apoptosis, reduced cell proliferation, decreased TNF-α, IL-6, and STAT3 levels, and alleviated DEN/HFD-induced hepatic steatosis and fibrosis, highlighting a key role for *TKT* in promoting genome instability during liver injury and tumor initiation 34. *CBFA2T2* (also known as *MTGR1*), a member of the Myeloid Translocation Gene (MTG) family of transcriptional corepressors, can significantly predict the survival of renal cell carcinoma (RCC) patients. Knocking-down of *CBFA2T2* can inhibit cell migration and invasion in RCC cells in vitro, and reduce ALDH high cancer stem cells (CSCs) populations. *CBFA2T2* expression is necessary for sphere-forming ability and cancer stem cells marker expression in RCC cell lines 35, 36. *CBFA2T2* is required for tumorigenesis in the murine colitis-associated carcinoma 37-39. *PAQR4* has a tumorigenic effect on human breast cancers, and such effect is associated with a modulatory activity of *PAQR4* on protein degradation of *CDK4* 40, 41. *PAQR4* promotes cell proliferation and metastasis in both non-small-cell lung cancer 42 and gastric cancer 43. *C21orf58* exerts a momentous effect on breast cancer cell growth 14. Nevertheless, the role of abnormal *CBFA2T2*, *PAQR4* or* C21orf58* in HCC remains undefined. Our study initially confirmed the negative effect of *CBFA2T2*, *PAQR4* or* C21orf58* on HCC prognosis for the first time. Conversely, tetratricopeptide repeat (TPR) domain protein 39B (TTC39B, C9orf52) (T39), a high density lipoprotein (HDL) gene discovered in human genome wide association studies (GWAS) 44, 45, is associated with atherosclerosis and steatohepatitis as well as inflammation 46. *HSPB3* is an unfavorable molecular biomarker in colorectal adenocarcinoma 47. Nevertheless, the role of *HSPB3* in HCC has not been illuminated.

Significantly, we formulated and identified a predictive prognostic model composed of seven genes to confer reference for HCC patient stratification in clinical practice. All enrolled HCC patients were sorted into high- and low-risk groups through mRNA expression levels rather than gene mutations or methylation alterations of merely seven prognostic genes. This method was more accessible and economical in practice considering that it diminished the utilization of whole-genome sequencing for all HCC subjects. Additionally, a nomogram was developed by combining this signature with conventional clinical indicators such as TNM stage, pathological differentiation, thus significantly enhancing the accuracy of predictive performance. It was also beneficial for clinicians to select high-risk HCC patients for adjuvant therapy except for surgical treatment. Notably, several limitations in our study need to be discussed. Initially, the clinical data from GEO database was insufficient and there was no additional valuable information concerning prognosis, including Child-Pugh scoring, cirrhosis scoring, AFP levels, tumor size and vascular invasion as well as therapeutic interventions. Furthermore, our study merely retrospectively analyzed relatively small sample size. A majority of patients from TCGA dataset were White or Asian. We should cautiously expand the results to additional ethnicities. Thirdly, further investigation should be warranted to determine the expression and the prognostic role of the seven genes at protein level as well as their underlying mechanisms. Thus, further independent prospective cohort studies with larger sample sizes and more elaborate clinical information are essential to validate the nine-gene signature and prognostic nomogram.

## Conclusion

A novel seven-gene signature for prognostic prediction in HCC was established, with higher risk scores implying unfavorable prognosis. A nomogram model integrating the seven-gene signature with additional significant clinicopathological parameters also yielded promising predictive performance in HCC survival.

## Supplementary Material

Supplementary figures and tables.

## Figures and Tables

**Table 1:** Basic characteristics of the seven genes in the prognostic signature

Gene stable ID	Gene name	Gene type	Chromosome	Gene Start (bp)	Gene end (bp)
ENSG00000163931	TKT	protein coding	3	53224707	53256114
ENSG00000155158	TTC39B	protein coding	9	15163622	15307360
ENSG00000198523	PLN	protein coding	6	118548296	118561716
ENSG00000078699	CBFA2T2	protein coding	20	33490070	33650031
ENSG00000169271	HSPB3	protein coding	5	54455699	54456377
ENSG00000162073	PAQR4	protein coding	16	2969348	2973484
ENSG00000160298	C21orf58	protein coding	21	46300426	46324046

**Table 2:** Univariate regression analysis of the seven genes significantly related to HCC OS

Genes	TCGA		GSE76427	
	HR (95% CI)	*p*	HR (95% CI)	*p*
TKT	1.2 (1.1-1.4)	0.0013	1.2 (1.1-1.4)	0.002
TTC39B	0.82 (0.72-0.94)	0.0039	0.76 (0.59-0.98)	0.032
PLN	0.94 (0.89-0.98)	0.0097	0.47 (0.27-0.84)	0.01
CBFA2T2	1.3 (1.1-1.7)	0.018	1.6 (1.1-2.4)	0.0074
HSPB3	0.96 (0.92-0.99)	0.025	0.65 (0.45-0.95)	0.026
PAQR4	1.2 (1-1.3)	0.026	1.3 (1-1.6)	0.027
C21orf58	1.2 (1-1.4)	0.037	1.3 (1.1-1.7)	0.018

**Table 3:** Association between the clinicopathologic parameters and the risk score levels in HCC

Variable	Groups	N	Low	%	High	%	HR (95% CI)	*p*
Age (years)	≤60	177	97	54.8%	80	45.2%	0.42 (0.25-0.74)	0.07
	>60	195	88	45.1%	107	54.9%	0.67 (0.43-1.1)	
Gender	Male	252	122	48.4%	130	51.6%	0.53 (0.34-0.83)	0.12
	Female	120	63	52.5%	57	47.5%	1.5 (0.86-2.71)	
Tumor Status	With	112	88	48.2%	96	51.8%	0.64 (0.38-1.1)	0.07
	Free	233	113	48.5%	120	51.5%	0.55 (0.33-0.92)	
	NA	27						
Race	White	184	88	47.8%	88	52.2%	0.71 (0.45-1.12)	0.12
	Asian	159	86	54.1%	73	45.9%	0.48 (0.27-0.88)	
	Black	17	7	41.2%	10	58.8%	2.76 (0.62-12.4)	
	NA	12						
BMI	≤26	204	109	53.4%	95	46.6%	0.48 (0.29-0.77)	0.09
	>26	151	66	43.7%	85	56.3%	0.66 (0.39-1.16)	
	NA	17						
Family history	No	209	110	52.6%	99	47.4%	0.37 (0.22-0.61)	0.99
	Yes	111	49	44.1%	62	55.9%	1.01 (0.57-1.75)	
	NA	52						
TNM	Stage I	172	77	44.8%	95	55.2%	0.63 (0.34-1.12)	0.12
	Stage II	86	44	51.2%	42	48.8%	0.63 (0.28-1.42)	
	Stage III	85	50	58.8%	35	41.2%	0.53 (0.30-0.96)	
	Stage IV	5	2	40.0%	3	60.0%		
	NA	24						
T classification	T1	182	82	45.1%	100	54.9%	0.69 (0.38-1.25)	0.22
	T2	94	51	54.3%	43	45.7%	0.70 (0.34-1.42)	
	T3	80	47	58.8%	33	41.3%	0.49 (0.27-0.86)	
	T4	13	5	38.5%	8	61.5%	0.25 (0.04-1.47)	
	TX	1	0	0.0%	1	100.0%		
	NA	2						
N classification	N0	253	129	51.0%	124	49.0%	0.62 (0.41-0.95)	0.02
	N1	4	4	100.0%	0	0.0%		
	NX	114	52	45.6%	62	54.4%	0.53 (0.29-0.98)	
	NA	1						
M classification	M0	267	136	50.9%	131	49.1%	0.61 (0.40-0.93)	0.01
	M1	4	1	25.0%	3	75.0%		
	MX	101	48	47.5%	53	52.5%	0.43 (0.23-0.80)	
Histologic grade	G1	55	14	25.5%	41	74.5%	0.44 (0.14-1.34)	0.04
	G2	178	84	47.2%	94	52.8%	0.61 (0.36-1.02)	
	G3	122	76	62.3%	46	37.7%	0.56 (0.31-1.02)	
	G4	12	8	66.7%	4	33.3%	0.79 (0.12-5.02)	
	NA	5						
Residual tumor	R0	325	156	48.0%	169	52.0%	0.48 (0.33-0.70)	0.13
	R1	17	14	82.4%	3	17.6%	2.52 (0.43-14.67)	
	R2	1	0	0.0%	1	100.0%		
	RX	22	12	54.5%	10	45.5%	4.0 (0.51-31.3)	
	NA	7						
AJCC staging	4th	4	0	0.0%	4	100.0%		
	5th	21	8	38.1%	13	61.9%	0.37 (0.11-1.20)	0.02
	6th	119	64	53.8%	55	46.2%	0.68 (0.41-1.12)	
	7th	228	113	49.6%	115	50.4%	0.68 (0.38-1.25)	
Child-Pugh	A	217	100	46.1%	117	53.9%	0.72 (0.22-2.26)	0.11
	B	21	10	47.6%	11	52.4%	0.97 (0.23-4.02)	
	C	1	0	0.0%	1	100.0%		
	NA	133						
Vascular invasion	None	207	94	45.4%	113	54.6%	0.53 (0.32-0.89)	0.48
	Micro	93	44	47.3%	49	52.7%	0.77 (0.35-1.68)	
	Macro	16	13	81.3%	3	18.8%	0.28 (0-38)	
	NA	56						
Virus infection	HBV	104	49	47.1%	55	52.9%	0.75 (0.32-1.86)	0.51
	HCV	53	26	49.1%	27	50.9%	0.52 (0.21-1.29)	
	No	169	88	52.1%	81	47.9%		
	NA	46						
Alcoholic	Yes	117	60	51.3%	57	48.7%	0.54 (0.29-1.02)	0.05
	No	225	110	48.9%	115	51.1%	0.6 (0.39-0.94)	
	NA	30						
AFP level	Normal	116	51	44.0%	65	56.0%	0.77 (0.35-1.71)	0.51
	Elevated	147	75	51.0%	72	49.0%	0.57 (0.33-1.0)	
	NA	109						
Fibrosis Score	0-4	133	60	45.1%	73	54.9%	0.55 (0.30-1.03)	0.03
	5-6	80	29	36.3%	51	63.8%	0.38 (0.11-1.35)	
	NA	159						
Hepatic Inflammation	No	117	52	44.4%	65	55.6%	0.57 (0.27-1.19)	0.08
	Mild	101	46		55	54.5%	0.54 (0.25-1.19)	
	Severe	17	7		10	58.8%	3.4 (0.32-36.3)	
	NA	137						
Relapse	No	173	84		89	51.4%	0.62 (0.31-1.21)	0.43
	Yes	98	48		50	51.0%	0.78 (0.42-1.47)	
	NA	101						

**Table 4:** Univariate and multivariate Cox regression analyses of the seven-gene signature and HCC OS in TCGA dataset

Variable	N	Univariate analysis		Multivariate analysis	
		HR (95% CI)	*p* value	HR (95% CI)	*p* value
**Age (years)**					
≤60	180	Reference			
>60	197	0.89 (0.63-1.3)	0.53		
**Gender**					
Female	120	Reference			
Male	252	1.5 (1.0-2.1)	0.03	1.2 (0.82-1.8)	0.33
**Race**					
Asian	159	Reference			
White	184	0.39(0.26-0.58)	0.55		
Black	17	0.55(0.24-1.24)	0.15		
NA	12				
**BMI**					
≤26	204	Reference			
>26	151	0.83 (0.58-1.2)	0.30		
NA	17				
**Family history**					
No	209	Reference			
Yes	111	0.83 (0.57-1.2)	0.32		
NA	52				
**TNM Stage**					
I	172	Reference			
II	86	0.99 (0.61-1.6)	0.98		
III	85	1.1 (0.71-1.6)	0.73		
IV	5	1.1 (0.34-3.6)	0.86		
**T classification**					
T1	182	Reference			
T2	94	1 (0.64-1.6)	0.94		
T3	80	1.2 (0.76-1.7)	0.50		
T4	13	1.5 (0.77-3.1)	0.22		
TX	1	0.95 (0.13-6.9)	0.96		
NA	2		0.53		
**N classification**					
N0	253	Reference			
N1	4	0.86 (0.21-3.5)	0.08		
NX	114	0.73 (0.51-1.1)	0.10		
NA	1				
**M classification**					
M0	267	Reference			
M1	4	1.1 (0.35-3.5)	0.85		
MX	101	0.92 (0.64-1.3)	0.68		
**Histologic grade**					
G1	55	Reference			
G2	178	1.1 (0.63-1.8)	0.01	1.5 (0.55-4)	0.04
G3	122	1.8 (1-3.2)	0.04	2.1 (0.73-5.8)	0.02
G4	12	2.6 (0.95-7.3)	0.06	4.2 (0.89-19)	0.07
NA	5				
**AJCC staging**					
4th	4	Reference			
5th	21	4.5 (1-20)	0.05	4.3 (0.95-20)	0.06
6th	119	6.5 (1.5-28)	0.01	4.9 (1.1-21)	0.04
7th	228	9.2 (2.1-40)	0.003	5.6 (1.2-25)	0.03
**Child-Pugh**					
A	217	Reference			
B	21	1.5 (0.71-3.2)	0.28		
C	1	0.45 (0.061-3.3)	0.43		
NA	132				
**Vascular invasion**					
Macro	16	Reference			
None	207	0.65 (0.31-1.4)	0.26		
Micro	93	0.73 (0.33-1.6)	0.43		
NA	56				
**Virus infection**					
HBV	104	Reference			
HCV	53	0.8 (0.42-1.5)	0.50		
No	169	0.86 (0.51-1.4)	0.56		
NA	46				
**AFP level**					
Elevated	147	Reference			
Normal	116	0.88 (0.54-1.4)	0.60		
NA	109				
**Fibrosis score**					
0-4	133	Reference			
5-6	80	1.6 (0.94-2.9)	0.04	1.2 (0.65-2.4)	0.51
NA	159				
**Risk score**					
High	185	Reference			
Low	187	0.55 (0.38-0.78)	0.0008	0.5 (0.27-0.94)	0.03

**Table 5:** Univariate and multivariate Cox regression analyses of the seven-gene signature and HCC OS in TCGA dataset

Variable	Univariate analysis		Multivariate analysis	
	HR (95% CI)	*p* value	HR (95% CI)	*p* value
**Age (years)**				
≤60	0.77 (0.33-1.8)	0.54		
>60	Reference			
**Gender**				
Male	1.2 (0.28-5.3)	0.79		
Female	Reference			
**BCLC Stage**				
A	Reference			
B	2.1 (0.83-5.3)	0.002	1.1 (0.45-2.5)	0.01
C	6.1 (1.8-21)	0.004	3 (0.78-12)	0.03
**TNM**				
I	Reference			
II	0.28 (0.036-2.2)	0.02	0.62 (0.26-1.5)	0.02
III	2.1 (0.27-17)	0.04	0.41 (0.086-1.9)	0.03
IV	0.19 (0.071-0.52)	0.001	2.3 (0.5-10)	0.001
**Risk score**				
Low	0.17 (0.063-0.43)	0.0003	0.15 (0.056-0.42)	0.0003
High	Reference			

## Abbreviations

HCC: hepatocellular carcinoma
TCGA: The Cancer Genome Atlas
GEO: Gene Expression Omnibus
DEmRNAs: Differentially expressed mRNAs
ROC: receiver operating characteristic
OS: overall survival
TNM: tumor-node-metastasis
AFP: alpha-fetoprotein
HR: hazard ratio
C-index: concordance index
AUC: under the curve
HBV: hepatitis B virus
HCV: hepatitis C virus
