# Supplementary material for: A novel seven-gene signature as Prognostic Biomarker in Hepatocellular Carcinoma
Source: J Cancer. 2020 Jul 31;11(19):5768–81. doi: 10.7150/jca.44573 (PMC7477431; doi:10.7150/jca.44573)

Supplementary Figure 1: The extraction of the seven common DEmRNAs. Heat map of DEmRNAs between HCC tissue and nonneoplastic tissues utilizing the (A) TCGA and (B) GSE47595 dataset. (C) Identification of the seven common DEmRNAs significantly associated with HCC prognosis through overlapping two independent datasets.

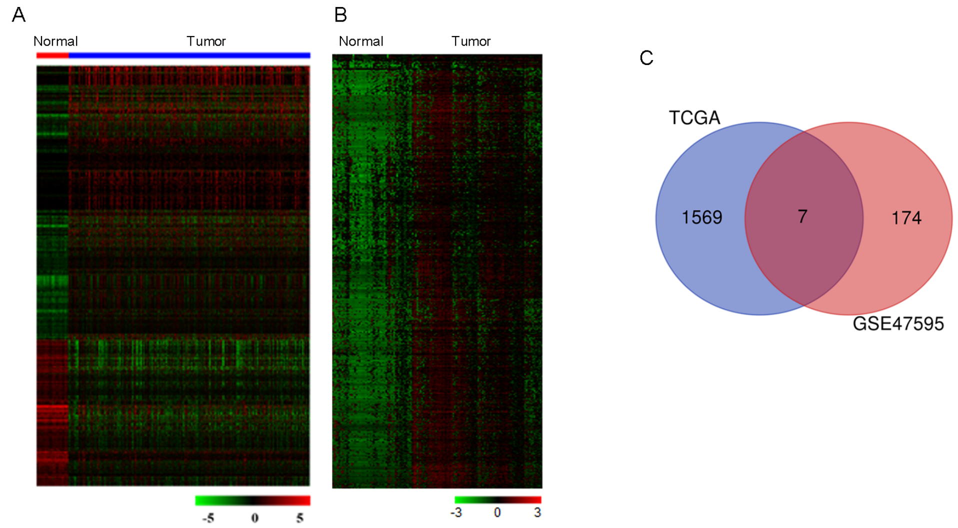

Supplementary Figure 2: Comparison of prognostic predictive accuracy between risk score model and other clinical parameters in (A) TCGA database and (B) GSE76427database.

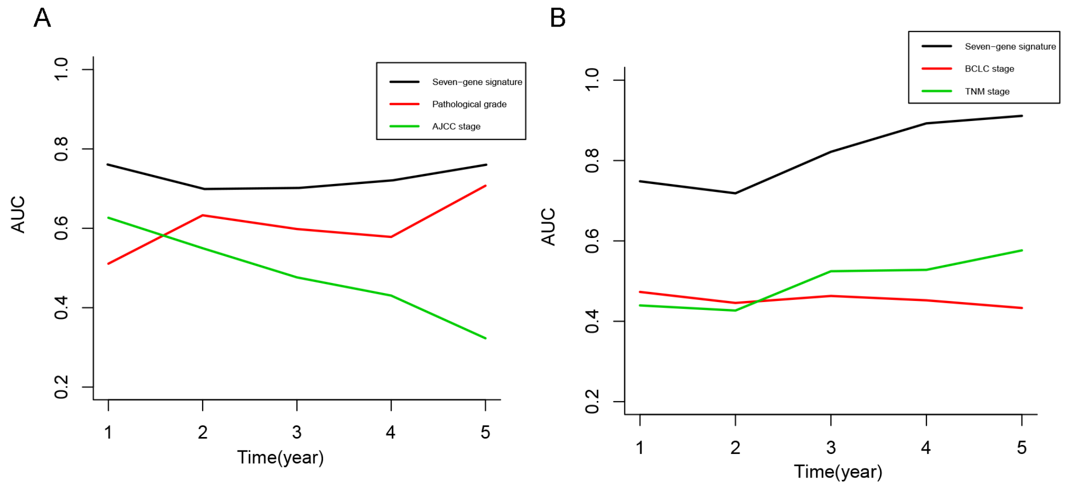

Supplement: Supplementary file 1 — Supplementary figures and tables. [file jcav11p5768s1.pdf]
